# Insights into Bacteriophage Application in Controlling *Vibrio* Species

**DOI:** 10.3389/fmicb.2016.01114

**Published:** 2016-07-19

**Authors:** Vengadesh Letchumanan, Kok-Gan Chan, Priyia Pusparajah, Surasak Saokaew, Acharaporn Duangjai, Bey-Hing Goh, Nurul-Syakima Ab Mutalib, Learn-Han Lee

**Affiliations:** ^1^Division of Genetics and Molecular Biology, Institute of Biological Sciences, Faculty of Science, University of Malaya, Kuala LumpurMalaysia; ^2^Novel Bacteria and Drug Discovery Research Group, School of Pharmacy, Monash University Malaysia, Selangor Darul EhsanMalaysia; ^3^Jeffrey Cheah School of Medicine and Health Sciences, Monash University Malaysia, Selangor Darul EhsanMalaysia; ^4^Center of Health Outcomes Research and Therapeutic Safety, School of Pharmaceutical Sciences, University of Phayao, PhayaoThailand; ^5^Pharmaceutical Outcomes Research Center, Faculty of Pharmaceutical Sciences, Naresuan University, PhitsanulokThailand; ^6^Division of Physiology, School of Medical Sciences, University of Phayao, PhayaoThailand; ^7^UKM Medical Molecular Biology Institute, UKM Medical Centre, Universiti Kebangsaan Malaysia, Kuala LumpurMalaysia

**Keywords:** bacterial, *Vibrio* sp., antibiotics, bacteriophages, multidrug resistant strains

## Abstract

Bacterial infections from various organisms including *Vibrio* sp. pose a serious hazard to humans in many forms from clinical infection to affecting the yield of agriculture and aquaculture via infection of livestock. *Vibrio* sp. is one of the main foodborne pathogens causing human infection and is also a common cause of losses in the aquaculture industry. Prophylactic and therapeutic usage of antibiotics has become the mainstay of managing this problem, however, this in turn led to the emergence of multidrug resistant strains of bacteria in the environment; which has raised awareness of the critical need for alternative non-antibiotic based methods of preventing and treating bacterial infections. Bacteriophages – viruses that infect and result in the death of bacteria – are currently of great interest as a highly viable alternative to antibiotics. This article provides an insight into bacteriophage application in controlling *Vibrio* species as well underlining the advantages and drawbacks of phage therapy.

## Introduction

The increased occurrence of foodborne disease has led to substantial morbidity and mortality around the world yearly, frequently associated with outbreaks or food contamination. Foodborne illness is known to be a ubiquitous, costly, yet preventable public health concern (Centers for Disease Control and Prevention [CDC], 2014). World Health Organization has stated that food safety remains an endless challenge to everyone particularly in the management of infectious and non-infectious foodborne pathogens (Rocourt et al., 2003). Despite the current effective technologies and the good manufacturing practices, the food safety is constantly threatened by the factors related to changes in lifestyle, consumer eating habits, food and agriculture manufacturing processes and also the increased international trade (Newell et al., 2010; Law et al., 2015).

There is no doubt that bacterial infection is a significant threat to mankind in many forms – human illness as a result of bacterial infection is common, with *Vibrio* species including *Vibrio cholerae*-associated from food contamination or transmission of infection from person to person, *Vibrio parahaemolyticus*- associated with food contamination and *Vibrio vulnificus*- associated with wound infection. *Vibrio* species are gram-negative curved rod shaped bacteria that belong to the *Vibrionaceae* family. They naturally inhabit the estuarine, coastal and marine environment worldwide (Letchumanan et al., 2014; Raghunath, 2015). The presence of this bacterium in the marine environment raises the concern of human on food safety due to the latter potential in causing disease outbreaks depending on the environmental conditions (Ceccarelli et al., 2013). There are many clinically used antibiotics as a choice of treatment for *Vibrio* species infections including cephalothin (first generation cephalosporins), cefuroxime (second generation cephalosporin), cefotaxime and ceftazidime (third generation cephalosporins), tetracycline, doxycycline, or fluoroquinolone (Tang et al., 2002; Han et al., 2007; Al-Othrubi et al., 2014).

Aside from this, these organisms have also been responsible for large scale losses in the aquaculture industry due to infection of the aquatic livestock leading to prophylactic as well as therapeutic use of antimicrobials (Devi et al., 2009; Manjusha and Sarita, 2011; Letchumanan et al., 2014, 2015b). In the Asian aquaculture industry, oxytetracycline, tetracycline, quinolones, sulphonamides, and trimethoprim are among the antimicrobials permitted and utilized to control bacterial infections (Rico et al., 2012; Yano et al., 2014). Our dependence on antibiotics to control bacterial infections in humans, aquaculture, agriculture, and veterinary medicine resulted to indiscriminate use which in turn led to the emergence of multidrug resistant strains in the biosphere (Rao and Lalitha, 2015).

Studies have reported the isolation of multidrug resistant *Vibrio* strains from both clinical and environmental samples (Letchumanan et al., 2015a; Shrestha et al., 2015; Zavala-Norzagaray et al., 2015). In Iran, *Vibrio cholerae* isolated from clinical samples has been reported to be resistant toward erythromycin, sulfamethoxazole-trimethoprim, and ampicillin (Tabatabaei and Khorashad, 2015). Antibiotic resistance was also observed in a study done in India which reported serogroups O1 of *Vibrio cholerae* classical biotype and sub serotype, Ogawa isolated from clinical strains were resistant to ampicillin, nalidixic acid, and cotrimoxazole (Shrestha et al., 2015). This bacterium is the causative agent of cholera and appears to be emerging as the etiological agent of disease outbreaks in many developing countries such as India (Garg et al., 2000), Bangladesh, Haiti (Sjölund-Karlsson et al., 2011), Vietnam (Tran et al., 2012), and Africa (Dalsgaard et al., 2001). *Vibrio cholerae* from clinical samples is reported to be resistant to many clinically used antibiotics including tetracycline (Roychowdhury et al., 2008), ampicillin (Petroni et al., 2002), nalidixic acid (Khan et al., 2015), streptomycin, sulfonamide, trimethoprim, gentamicin (Dalsgaard et al., 1999), and ciprofloxacin (Khan et al., 2015).

A study in Thailand has revealed that shrimp farmers were highly dependent on various antibiotics as a preventive measure against shrimp bacterial infections with 14% of farmers using antibiotics on a daily basis in their farms (Holmstrom et al., 2003). In Malaysia, *Vibrio parahaemolyticus* strains isolated from seafood and environmental sources were reported to be resistant toward cefalexin and ciprofloxacin (Al-Othrubi et al., 2011). Besides, antibiotic resistant *Vibrio parahaemolyticus* strains have been isolated from both clinical and environmental samples in India (Pazhani et al., 2014; Reyhanath and Kutty, 2014; Sudha et al., 2014). A study assessed the diversity of antibiotic resistant bacteria and their resistance genes from mariculture environments of China. It was reported that the strains exhibited multidrug resistance profile toward oxytetracycline, chloramphenicol, and ampicillin (Dang et al., 2007). Frequent use of antibiotics is also widely apparent in other regions such as Mexico (Roque et al., 2001), Philippines (Tendencia and De La Pena, 2001), Italy (Lalumera et al., 2004), Malaysia (Al-Othrubi et al., 2011; Sani et al., 2013; Letchumanan et al., 2015b), Thailand (Yano et al., 2014), and China (Peng et al., 2010; Zou et al., 2011; Xu et al., 2014). The various antibiotics used in aquaculture has led to the occurrence of antibiotic resistant genes (ARGs) in bacteria. Many different ARGs can be found in bacteria in the environment. For example, β-lactam and penicillin resistant genes *penA* and *blaTEM-1* (Srinivasan et al., 2005; Zhang et al., 2009), chloramphenicol resistant genes *cat*I, *cat*II, *cat*III, *cat*IV, and *floR* (Dang et al., 2007, 2008), tetracycline resistant genes *tatA, tatB, tatC, tatD, tatE, tatG, tatH, tatJ, tatY, tatZ*, and many more (Macauley et al., 2007; Zhang et al., 2009, 2012; Kim et al., 2013). It is reported that ARGs could be transferred among bacteria via conjugation, transduction, or transformation (Manjusha and Sarita, 2011).

The widespread of emergence of antimicrobial resistant bacteria worldwide has become a major therapeutic challenge (Giamarellou, 2010). There is need for development of novel non-antibiotic approach to fight against bacterial infections due to the shortage of new antibiotics in developmental pipeline (Rice, 2008; Freire-Moran et al., 2011). Recently, there has been renewed interest in the application of bacteriophage as a non-antibiotic approach to control bacterial infections in various fields including human infections, food safety, agriculture, and veterinary applications (Wittebole et al., 2014). This article provides an insight into bacteriophage application in controlling *Vibrio* species as well underlining the advantages and drawbacks of phage therapy.

## Bacteriophages

### Historical Background

Early discovery of bacteriophage was reported by M. E. Hankin in 1896 after observing antibacterial properties of this viral-like agent against *Vibrio cholerae* in Ganges River, India (Adhya and Merril, 2006). The phage’s nature was clearly defined following the observation of its capability of lysing bacterial cultures by Frederick Twort and Felix d’Herrelle, in 1915 and 1917, respectively (Adhya and Merril, 2006). It was Felix d’Herrelle who named this viral-like agents as bacteriophage and implemented in the treatment of human diseases almost instantly after their discovery. Bacteriophage therapy appeared as the frontline therapeutics against infectious disease before the discovery of the broad spectrum antibiotic and were used in various countries until The Second World War (Enderson et al., 2014). Unfortunately, the use of phages as therapeutic agents and phage research declined due to the limited knowledge of phage properties, contradictory results from various published studies and discovery of antibiotics (Wittebole et al., 2014). However, over the last decade, the therapeutic value of bacteriophage has been reconsidered due to the occurrence of multi-drug resistant bacteria. Bacteriophages are regarded as an alternative non-antimicrobial tool to treat bacterial infections while controlling the emergence of antibiotic resistance (Meaden and Koskella, 2013; Payet and Suttle, 2014). The research into phage therapy has been further encouraged given that regulatory bodies in charge of food safety have approved the utilization of certain phages for use in food products such as ListShield^TM^, and Listex P100 (Bren, 2007; Coffey et al., 2010). ListShield^TM^, (a phage which targets *Listeria*) from Intralytix is approved by the USFDA for the treatment of food products, and the phages are classified as Generally Recognized As Safe (GRAS; FDA, 2013).

### Morphology

Phages are bacterial viruses that are able to infect bacterial host cells with high host specificity of strain or species level (Hagens and Loessner, 2010) and subsequently multiply, eventually resulting in death of the host cell. While high host specificity is typical, a few phages do exhibit wide host ranges and are able to infect a large subset of a given species or even multiple species (Chen and Novick, 2009). Bacteriophages species can be differentiated as they vary both in size 24–400 mm in length and genome length. All bacteriophages have a head that stores genetic materials and form a part of the overall feature of a bacteriophage (Orlova, 2012). Structurally, a phage consist of a core nucleic acid encapsulated with a protein or lipoprotein capsid which is connected with a tail that interacts with various bacterial surface receptors via the tip of the tail fibers. This interaction shows an affinity that is specific to a certain group of bacteria or even to a particular strain (Deresinski, 2009; Tan et al., 2014). The capsid is icosahedral in shape and has the main function to protect the genetic material from the environment. A bacteriophage head is attached to a tail through a connector that functions as adaptor between these two structures of the phage. The tail is a hollow tube which acts as a passage way for genetic materials to pass thru from capsid to host bacteria (Lurz et al., 2001). Tail fibers and base plate which are located at the end structure of the phage are involved in the binding process of the phage to the bacterial outer membrane (Sao-Jose et al., 2006).

### Nature of Bacteriophages

Bacteriophages are the most abundant organisms in the environment, with the total number of phages on Earth estimated to be between 10^30^ and 10^31^, an approximately 10 times more than their bacterial hosts (Abedon et al., 2011; Burrowes et al., 2011). Phages are natural predators of bacteria, self-limiting and self-replicating in their host cell, and can adapt to resistant bacteria (Carvalho et al., 2010; Jaiswal et al., 2014). They are commonly found in large numbers wherever their hosts live; in sewage, in soil, in hatchery, in deep thermal vents, or in natural bodies of water (Karunasagar et al., 2007; Kim et al., 2012; Rong et al., 2014). To date, most of the marine viruses reported are bacteriophages that belong to order *Caudovirales*, which is divided into three families: *Siphoviridae* (icosahedral capsid with filamentous non-contractile tail), *Myoviridae* (icosahedral symmetrical head with a helical contractile tail separated by neck) and *Podoviridae* (icosahedral symmetrical head with very short non-contractile tail; Suttle, 2005; Rao and Lalitha, 2015).

### Genetic and Genome of Phages

Bacteriophages are viruses with either DNA or RNA as their genetic material. They appear in both single and double stranded forms. The structure is similar to the living organisms found in the environments; with a polynucleotide chain consisting of a deoxyribose (or ribose) phosphate backbone to which are attached to a specific sequence of the four nucleotides – adenine, thymine (or uracil), guanine, and cytosine. It is exceptional in single stranded phages where two complementary chain are paired together in a double helix (Benett and Howe, 1998).

The complete genome sequence of the T4-like phage, vibriophage KVP40 has been studied in Japan. This vibriophage belong to *Myoviridae* family has a double-stranded DNA genome sequence in length of 244,835 bp, a prolate icosahedral capsid, and a contractile tail with associated baseplate and extended tail fibers. KVP40 has a very broad host range covering several species of *Vibrio* including *Vibrio cholera, Vibrio anguillarum, Vibrio parahaemolyticus*, and the non-pathogenic species *Vibrio natriegens*, and *Photobacterium leiognathi*. The presence of several copies of genes encoding proteins linked with phage tail or tail fibers in the KVP40 genome suggest an increased flexibility in host range adaptation (Miller et al., 2003). Another genome study reported on Phage vB_VpaM_MAR isolated from fresh non-treated seawater samples in Mexico. Phage vB_VpaM_MAR belongs to *Myoviridae* family has a high specificity to host, able to lyse 76% of the *Vibrio parahaemolyticus* strains tested. Sequence analysis shows the genome of phage MAR is 41,351 bp double-stranded DNA with a G+C content of 51.3% and encodes 62 open reading frames (ORFs; Villa et al., 2012). A novel *Vibrio vulnificus*-infecting bacteriophage, SSP002, belonging to the *Siphoviridae* family, was isolated from the coastal area of the Yellow Sea of South Korea. Host range analysis revealed that the growth inhibition of phage SSP002 is relatively specific to *Vibrio vulnificus* strains from both clinical and environmental samples. A comparative genomic analysis of phage SSP002 and *Vibrio parahaemolyticus* phage vB_VpaS_MAR10 showed differences among their tail-related genes, supporting different host ranges at the species level, even though their genome sequences are highly similar (Lee et al., 2014). Recently, two broad-host range phage (H1 and H7) were isolated from Danish fish farms. Both these phages belong to the *Myoviridae* family and had large genome size (194 kb; Demeng et al., 2014). Interestingly, vibriophage genome provides a detailed characterization on the phage properties as well as understanding of the phage host range and interaction. This information is essential in order to overcome the drawbacks of phage therapy and ensure successful phage application.

### Life Cycle of Phages

As natural viruses of bacteria, phages their infection in their bacterial host by reversible adsorption to the specific host cell via specific cell-surface proteins. They then eject their genetic material into the cytoplasm of the bacterial host (Burrowes et al., 2011; Molineux and Panja, 2013). Bacteriophages have two apparent lifecycles; the lytic cycle and lysogenic cycle. The lytic cycle is a form of infection which results in direct damage to the bacterial host. It involves a series of events that occur between attachment of phage particle to a bacterial cell and release of daughter phage particles. There are four phases in the lytic cycle; the adsorption of phage to host cell by binding to specific host, penetration of phage nucleic acid, intracellular development and finally destruction of the cell wall, releasing the newly assembled phages into the environment. In detail, after binding and injection of phage genome into the host cell, the virulent bacteriophages will control the host cell’s protein machinery via the expression of specific enzyme encoded by phage genome. It redirects the bacterial synthesis machinery to reproduction of the new phage particles. The production of phage’s enzyme in the later stage such as lysins and holins induce destruction of the cell membrane allowing the newly formed phages burst out from the lysed host cell into the environment (Young, 1992). This entire process takes about 20 min to 2 h (Rao and Lalitha, 2015).

The lysogenic cycle, by contrast, involves the replication of phage nucleic acid along with host genes for several generations without major destruction to the host cell. It is a latent mode of infection which happens in a very low frequency (Cochran et al., 1998). The phage genome remains in a repressed state in the host genome and is replicated as part of the bacterial chromosome until lytic cycle is induced. Hence, temperate phages are not suitable for direct therapeutic use as it may mediate transduction by transferring genetic material of one bacterium to another. This process may lead to the development of antibiotic resistance or even increased virulence of the host by acquiring genes from the prophage. Lytic bacteriophages which replicate exponentially and destroy the bacterial host regardless of their antibiotic resistance profile, are more suitable for the biotherapy purposes (Sillankorva et al., 2012).

## Phage Therapy

Bacteriophages have been used in many countries since 1929 – before the discovery of broad spectrum antibiotics – as a therapeutic agent against infectious disease (Tan et al., 2014). The first bacterium tested against bacteriophage therapy was *Vibrio cholerae* but the phage activity was reported to be higher *in vitro* compared to *in vivo* (Adams, 1959). The clinical use of phages as therapeutic agents and phage research started to decline and eventually ceased due to the limited knowledge of phage properties and contradictory results from various published studies. The therapeutic use of bacteriophages was further reduced after the emergence of antibiotics (Tan et al., 2014) although phage research and development still remained active in former Soviet Union and Poland (Sulakvelidze et al., 2001). Interestingly, the therapeutic value of bacteriophages has been reevaluated over the most recent decade because of the rise of multidrug resistant bacteria.

*Vibrio* sp. such as *Vibrio harveyi*, *Vibrio parahaemolyticus*, *Vibrio campbellii* are known to be the causative agent of luminous vibriosis disease in shrimp farm. This has resulted in 50–100% mortality rate among shrimps and cause of *Vibrio* infection in human (Shruti, 2012; Letchumanan et al., 2014; Wang et al., 2015; Tan et al., 2016). Bacteriophages isolated from hatchery water have proven to be effective in controlling luminous vibriosis disease, suggesting the phage’s potential as a biocontrol agent for luminous vibriosis (**Table 1**). Vinod et al. (2006) reported the isolation and trial of a phage that has potential to control population of pathogenic *Vibrio harveyi* in a hatchery setting. The study isolated a double stranded DNA bacteriophage of *Vibrio harveyi* belonging to the family *Siphoviridae* from shrimp water farm from the west coast of India. The application of phage to control luminescent vibriosis of shrimp larvae was tested in laboratory and hatchery trial. In the laboratory microcosm, a set up containing post larvae of *Penaeus monodon* was exposed to *Vibrio harveyi* and the level of the pathogen was around 10^6^ cfu ml^-1^. The treatment with 100 ppm phage twice has led to two log reduction of *Vibrio harveyi* counts. Larval survival without treatment was only 25% at 48 h but 80% with treatment with two doses of bacteriophage. In the hatchery trial setting, three sets of 500 L tanks containing 35,000 nauplii of *Penaeus monodon* was reared for 17 days. The antibiotic treated tanks (treated with oxytetracycline 5 ppm and kanamycin 100 pm daily) resulted to initial reduction of luminous bacterial counts but after 48 h, the disease appeared again and proliferated to a level of about 10^6^ ml^-1^. While in the tanks treated with bacteriophage, luminous bacteria were not detected throughout the 17-day study period. The luminous bacteria proliferating in control tanks appeared to be virulent, causing mortalities in the larvae. Overall, the survival rate in control tank was only 17%, while in antibiotic treated tanks it was 40% and in the bacteriophage treated tank, it was 86%. Vinod et al. (2006) concluded that since there is a ban on the use of most antibiotics in aquaculture, bacteriophages have the potential to manage luminous vibriosis in the aquaculture setting.

**Table 1:** Potential application of bacteriophage therapy for *Vibrio* species in aquaculture.

Aquaculture product	Etiologic agent	Bacteriophage	Bacteriophage source	Bacteriophage Administration	Results	Reference
Shrimp larvae *Penaeus monodon*	*Vibrio harveyi*	*Myoviridae* (VHLM)	Extracted from a toxin-producing strain of *Vibrio harveyi* isolated from moribund prawn larvae		VHML showed a narrow host range and an apparent preference for *Vibrio harveyi* rather than other 63 *Vibrio* isolates and 10 other genera.	Oakey and Owens, 2000; Oakey et al., 2002

Shrimp larvae *Penaeus monodon*	*Vibrio harveyi*	*Siphoviridae*	Isolated from shrimp farm water from West coast of India	Eighteen day old shrimp were challenged with the bacteria (10^5^ cells ml^-1^). Laboratory trials: (1) Bacteriophage suspension 10^9^ pfu ml^-1^ were added initially and another 0.1 ml after 24 h. (2) 0.1 ml of phage suspension. (3) No addition. Hatchery trial: in triplicates (1) Treatment with 10^9^ pfu ml^-1^ bacteriophage at the rate of 200 ppm daily so the phage concentration in the water is 2 × 10^5^ pfu ml^-1^ (2) Treatment daily with 5 ppm oxytetracycline and 10 ppm kanamycin. (3) No treatment.	The laboratory trial showed that survival of *Penaeus monodon* larvae was enhanced 80% with treatment with two doses of bacteriophage as compared with the survival rate in control was only 25%. In the hatchery trial, survival in control tank was only 17% while in antibiotic treated tanks was 40%. In the bacteriophage treated tank, the survival rate was 86%. Conclusion: The study concluded that bacteriophage has the potential in management of luminous vibriosis in aquaculture.	Vinod et al., 2006

Shrimp larvae *Penaeus monodon*	*Vibrio harveyi*	Lytic bacteriophages against *Vibrio harveyi*, two from *Siphoviridae*	Three isolated from oyster tissue and one from shrimp hatchery water.	Tanks with post larval five stage larvae, showing luminescence and mortality were used. Two tanks were treated with bacteriophage: one suspension (2 × 10^6^pfu ml^-1^) was added by day following the order: Viha10, Viha8, Viha10, and Viha8. Two tanks were treated with 5 mg L^-1^ of oxytetracycline and 10 mg L^-1^ of kanamycin	Over 85% survival of *Penaeus monodon* larvae after bacteriophage treatment. The normal hatchery practice of antibiotic treatment could only result 65–68% of survival. Conclusion: The study showed that bacteriophages could be used for biocontrol of *Vibrio harveyi*.	Karunasagar et al., 2007

Penaeid shrimp	*Vibrio harveyi*	Bacteriophage specific to *Vibrio harveyi* (Viha1 to Viha7), six from *Siphoviridae*, and one from *Myoviridae* (Viha4)	Isolated from coastal aquaculture systems like shrimp farms, hatcheries, and tidal creeks along the East and West coast of India		All the phage were found to be highly lytic for *Vibrio harveyi* and had different lytic spectrum for the large number of isolates tested. Three of the phages (Viha1, Viha3, and Viha7) caused 65% of the strains to lyse while Viha2, Viha4, and Viha6 caused 40% of the host strains to lyse. Viha5 had a narrow spectrum (14%). Conclusion: Six of the seven phages isolated had a broad lytic spectrum and could be potential candidates for biocontrol of *Vibrio harveyi* in aquaculture systems.	Shivu et al., 2007

Shrimp	*Vibrio harveyi*	*Siphoviridae* (VH1 to VH8)	Isolated from shrimp farm	*In vitro* experiments	All the isolates of bacteriophage caused lysis of the host bacterial cells within 2 h. The propagation curve for each phage shows a burst time started from 1 to 10 h. Conclusion: Bacteriophage of *Vibrio* species could be effectively used *in vivo* as biological agents to control these pathogenic bacteria in aquaculture systems.	Srinivasan et al., 2007

Shrimp	*Vibrio harveyi* CS101	*Siphoviridae* (Phage PW2)	Isolated from shrimp pond water		The phage adsorption rate increased rapidly in the 15 min of infection to 80% and continued to increase to 90% within 30 min of infection. The stability of phage PW2 was dependent on temperature and pH. It was inactivated by heating at 90°C for 30 min and by treating at pH 2, 3, 11, and 12. From its one step growth curve, latent, and burst periods were 30 and 120 min, respectively, with a burst size of about 78 pfu per infected center. Six structural proteins were detected.	Phumkhachorn and Rattanachaikunsopon, 2010

Phyllosoma larvae of the tropical rock lobster *Panulirus ornatus*	*Vibrio harveyi*	Six bacteriophages from *Siphoviridae* (VhCCS-01, VhCCS-02, VhCCS-04, VhCCS-06, VhCCS-17, and VhCCS-20) and two from *Myoviridae* (VhCCS-19, VhCCS-21)	Isolated from water samples of discharge channels and grow-out ponds of a prawn farm	Bacteriophage treatments in triplicate: (1) Addition of 1 ml of VhCCS-06 phage at 2 h after inoculation. (2) Addition of 1 ml of VhCCS-06 phage at 6 h after innoculation.	The *Myoviridae* (VhCCS-19 and VhCCS-21) were lysogenic and appeared in a limited number of host bacteria. The *Siphoviridae* phage (VhCCS-06) delays the entry of a broth culture of *Vibrio harveyi* strain 12 into exponential growth but could not prevent the overall growth of the bacterial strain. This effect was most likely due to multiplication of phage-resistant cells.	Crothers-Stomps et al., 2010

Oysters	*Vibrio parahaemolyticus*	*Siphoviridae* pVp-1	Isolated from coastal water of the Yellow Sea, Korea	Oysters infected with *Vibrio parahaemolyticus* were treated with bacteriophage by bath immersion and surface application.	After 72 h of phage application with bath immersion, bacterial growth reduction was observed at 8.9 × 10^6^ CFU/ml (control group) to 1.4 × 10 CFU/ml (treatment group). Bacterial growth was properly inhibited in the surface-applied group. After 12 h of phage application on surface of oysters, bacterial growth inhibition was revealed to be 1.44 × 10^6^ CFU/ml (control group) to 1.94 CFU/ml (treatment group).	Jun et al., 2014a

Oysters	*Vibrio parahaemolyticus*	Lytic phage VPp1	Isolated from sewage samples	Oysters were infected with 10^5^, 10^6^, 10^7^ CFU/ml of *Vibrio parahaemolyticus* and each infected group was treated with three different MOI values: 10, 1, and 0.1. at 22, 20, 16, and 12°C for 36 h.	The temperatures <20°C were safe for oyster rearing. Depuration at 16°C with 0.1 MOI was the best condition for reducing *Vibrio parahaemolyticus* in oysters, which decreased by 2.35–2.76 log CFU/g within 36 h.	Rong et al., 2014

Fish	*Vibrio anguillarum*	Lytic phage	Isolated from bivalves	**(a) In laboratory:** Three tank conditions, one tank for each condition, as follows: Groups of 15 *S. salar* of 8–25 g per tank (100 L) were maintained in aerated dechlorinated freshwater and a water recirculation system. A fresh culture of the *Vibrio anguillarum* strain PF4 was added directly to the water to a final concentration of 5 × 10^5^ CFU/mL, while an equal volume of fresh medium was added to the control tank. The phage was added directly to the water immediately after the addition of the bacteria **(b) In fish farm facilities:** Performed with groups of 100 *S. salar* of 20–25 g per tank (250 L). The fish were maintained at 12–15°C, in fresh water that was adjusted to 1% of salinity with seawater and in the presence of a water recirculation system that exchanged 50% of the water every day. The tank conditions were set up as the conditions as previous with the addition of one tank condition to test the effect of the phage alone on the fish. In this tank, the phages were added at the same concentration as in the tank with bacteria and phage.	The presence of the phage increased the survival of fish to 100% when it was used with a MOI of 1 and 20, versus less than 10% of survival in the absence of the phage.	Higuera et al., 2013

An *in vivo* study utilizing bacteriophage to control luminous vibriosis has further proven phage’s potential as a biocontrol agent of luminous vibriosis in aquaculture (Karunasagar et al., 2007). The study isolated four bacteriophages; Viha9, Viha10, Viha11 from oyster, and Viha8 from hatchery water. The morphological characteristic of both phage Viha8 and Viha10 had a non-contractile tail and contained double stranded DNA, hence both the phages were confirmed as members of *Siphoviridae*. Both Viha8 and Viha10 were subjected to laboratory trial and hatchery trials. The results from the laboratory trials revealed that phage Viha10 was able to lyses 70% of *Vibrio harveyi* strains tested while Viha8 had the ability to lyses 68% of the *Vibrio harveyi* strains. The *Vibrio harveyi* strains that were not able to be lysed by Viha10 were lysed by Viha8, and thus using this combination of Viha8 and Viha10, 94% of the *Vibrio harveyi* strains tested were lysed. Hence, Karunasagar et al. (2007) suggested the use of Viha8 and Viha10 combination as the biocontrol for *Vibrio harveyi* in hatchery trials. In the hatchery trials, four tanks (A, B, C, and D) of *Penaeus monodon* larvae were infected with *Vibrio harveyi* as evidenced by luminescence. Tank A and B were treated with both phage Viha8 and Viha10 alternately; first day with bacteriophage Viha10 at a level of 2 × 10^6^ pfu ml^-1^ and the following day, phage Viha8 was used at the same concentration. This treatment regimen was repeated on the third day with Viha10 and fourth day with Viha8. On the other hand, tank C was treated with oxytetracycline and tank D with kanamycin. The results showed that the survival rate of *Penaeus monodon* larvae in bacteriophage treated tank was 86–88% while the antibiotic treated tanks was 65–68% survival rate. The study concluded bacteriophages were effective in controlling luminous vibriosis in hatchery settings (Karunasagar et al., 2007).

Further interest on bacteriophage’s potential as a biocontrol agent has led to the discovery and isolation of a novel phage in Korea. Phage pVp-1 was isolated from the coastal water of Yellow Sea in Korea demonstrated efficiency in controlling *Vibrio* species (Kim et al., 2012). In addition, this novel marine siphovirus was also reported to be effective infecting *Vibrio parahaemolyticus* ATCC33844, a clinical strain isolated from patient with food poising in Japan (Kim et al., 2012). Jun et al. (2014b) demonstrated how phage pVp-1 was utilized against a multiple-antibiotic resistant *Vibrio parahaemolyticus* pandemic strain, CRS 09-17. In the study oysters infected with CRS09-17 strain was treated with pVp-1 by bath immersion and surface application. The two different method of phage treatment was applied considering the oysters processing; oysters infected model of *Vibrio parahaemolyticus* encountered during aquaculture or fishery markets; and second, the oysters surface contamination model of *Vibrio parahaemolyticus*, which are commonly encountered at restaurants. After 72 h of phage application with bath immersion, bacterial growth reduction was observed to be 8.9 × 10^6^ CFU/ml (control group) to 1.4 × 10 CFU/ml (treatment group). When pVp-1 was surface-applied on the flesh of oysters after CRS 09-17 inoculation, bacterial growth was properly inhibited. After 12 h of phage application on the surface of oysters, bacterial growth inhibition was revealed to be 1.44 × 10^6^ CFU/ml (control group) to 1.94 CFU/ml (treatment group). Overall, the phage application to various aquaculture situation emphasizes the potential use of the phage to avoid *Vibrio parahaemolyticus* infection from aquaculture to consumption (Jun et al., 2014b).

The *Siphoviridae* phage pVp-1 was used in another *in vivo* study by Jun et al. (2014a) involving mice infected with *Vibrio parahaemolyticus*. The efficacy of phage therapy was evaluated in two experiments using the *Vibrio parahaemolyticus* CRS 09-17 infection mouse model. In the first experiment, two groups of mice (control/treatment; five mice in each group) were challenged by an IP injection of an LD_50_ of CRS 09-17. Each mouse was treated with a single IP injection of phage pVp-1 (2.0 × 10^8^ PFU per mouse) or PBS 1 h after the bacterial challenge (2.0 × 10^7^ CFU per mouse). In the second experiment, all conditions were similar to those of the first study except that the bacterial challenge (2.0 × 10^7^ CFU per mouse) and phage treatment (2.0 × 10^8^ PFU per mouse) were administered orally. Both experiments were repeated five times, and the health of the mice was monitored for 72 h. In an additional study, two groups (five mice per group) were not challenged with bacteria and received only phage (2.0 × 10^11^ PFU per mouse) by IP and oral routes. The health of these mice was monitored for 28 days. The study concluded that phage-treated mice exhibited from a *Vibrio parahaemolyticus* infection and survived lethal oral and intraperitoneal bacterial challenges (Jun et al., 2014a).

Rong et al. (2014) reported the effectiveness of phage VPp1 application to reduce the population of *Vibrio parahaemolyticus* in the oyster depuration. VPp1, a lytic phage that was isolated from sewage was capable of reducing *Vibrio parahaemolyticus* infection on oysters by 2.35–2.76 log cfu/g within 36 h (Rong et al., 2014). Another study isolated a lytic phage named as PW2 from shrimp pond water in Songkhla Province, Thailand. The morphological characteristics showed that this phage has an icosahedral head and a long non-contractile tail, which can be categorized under the order Caudovirales and family of *Siphoviridae*. This phage PW2 showed lytic properties against *Vibrio harveyi*. Based on previous studies, most of the *Vibrio harveyi* phages were found to be siphophages with double stranded DNA (Pasharawipas et al., 2005; Vinod et al., 2006; Karunasagar et al., 2007; Jun et al., 2014a,b). However, *Vibrio harveyi* phages from other families such as Myoviridae and Podoviridae were also reported (Oakey and Owens, 2000; Oakey et al., 2002; Shivu et al., 2007).

Cholera, a water borne disease continues to be a major public health concern in developing countries and re-emerging in countries where it disappeared long time ago (World Health Organization [WHO], 2008). The occurrence of multidrug antibiotic resistant strains of *Vibrio cholerae* in the environment has prompted the search of alternative source of treatment such as bacteriophage therapy. The usefulness of lytic cholera phage as a prophylactic agent has been studied in many countries (Monsur et al., 1970; Marcuk et al., 1971). Jaiswal et al. (2013) studied the efficacy of five lytic vibriophage cocktail in treating *Vibrio cholerae 01* biotype El Tor serotype Ogawa MAK 757 (ATCC 51352) infection in rabbit model. It was observed that oral administration of phage cocktail after oral bacterial administration reduced the shedding of bacteria significantly (*p* < 0.01). The rabbits appeared normal without any toxicity evidence. The study concluded that phage cocktail was more potent as a lytic agent compared to as individual phages. An oral administration of suitable phage cocktail would be suitable as an alternative to antibiotic treatment in case of cholera infection (Jaiswal et al., 2013).

An oral phage cocktail (ATCC- B1, B2, B3, B4, B5) was administrated in adult mice model in a study by Jaiswal et al. (2014). The study performed a comparative analysis between phage cocktail, antibiotic, and oral rehydration treatment for orally developed *Vibrio cholerae* infection. It was reported that the genome size of vibriophage B1, B2, B3, B4 was around 40 kb and phage B5 had a genome size of around 100 kb. *In vitro* characteristic of vibriophages showed theses phages could withstand variety of pH level (pH 2–pH 12) as well as temperature range of 25–60°C. The study reported that combination of five vibriophages cocktail reduced the number of *Vibrio cholerae* cells in the orally infected mice compared to antibiotic and oral rehydration treatment (Jaiswal et al., 2014). A previous study analyzed the usefulness of phage cocktail in a *Vibrio cholerae 01* infected RITARD (removable intestinal tie-adult rabbit diarrhea) model experiment. The study concluded that cocktail of phage could provide significant protection and act as prophylaxis against *Vibrio cholerae* infection (Bhowmick et al., 2009).

In general, the selection of appropriate bacteriophage is a key factor in the success of phage therapy of *Vibrio* species (Mateus et al., 2014). Based on the studies discussed, bacteriophage belonging to *Siphoviridae* family is selected to control *Vibrio* species. *Siphoviridae* phage is reported to have a specific host range and closely related to species of *Vibrio harveyi*, *Vibrio parahaemolyticus*, and *Vibrio campbellii* (Crothers-Stomps et al., 2010). In addition phage cocktails have been demonstrated to be more effective than individual phages in treatment of *Vibrio cholerae* infection (Bhowmick et al., 2009; Jaiswal et al., 2014). By making a phage cocktail, it would become easier to treat a wide range of drug-resistant bacterial infections (Golkar et al., 2014). Although bacteriophages are isolated from different environmental source such as shrimp hatchery, sewage, ponds or from aquatic animals, they still show the same bacteriolytic activity and possess advantages over conventional antibiotics (Gutierrez et al., 2010).

## Advantages of Phage Therapy

Bacteriophages are natural antibacterial agents that are able to regulate bacterial populations by inducing bacterial lysis. Phages are reported to be active against both Gram-negative and Gram-positive bacteria including multidrug resistant pathogens in the environment (Biswas et al., 2002; Matsuzaki et al., 2003; Wang et al., 2006; Vinodkumar et al., 2008; Wittebole et al., 2014). Bacteriophages have a number of desirable properties that make them compelling candidates for tackling antibiotic resistance in bacteria. A bacteria is unable to regain its viability after been lysed by lytic phage; by contrast antibiotic therapy may not kill the targeted bacteria, facilitating the development of antibiotic resistance (Stratton, 2003).

The high specificity for their host cell is another advantage of bacteriophages relative to antibiotics. Phages are very specific to their host thus reducing the chances of secondary infections. They do not affect or alter the gut microbiota nor change the organoleptic properties of food products (Hagens and Offerhaus, 2008). Phages are specific bacterial host killers and do not affect normal microbiota compared to antibiotics which affect bacterial cells non-selectively (Rao and Lalitha, 2015). Additionally, there is no adverse effects reported during or after the phage treatment whereas allergies, secondary infections and bacterial resistance are common side effects seen after antibiotic treatments (Sulakvelidze et al., 2001).

Phages have the capability to replicate selectively at the site of infection where they are needed to lyse their bacterial hosts in contrast to antibiotics which distribute throughout the body fluids and tissues based on their inherent pharmacokinetic properties rather than becoming concentrated at the site of infection (Golkar et al., 2014). Phages are very environmentally friendly and evolved based on natural selection. Isolating and identifying suitable phages for therapy is a relatively simple, rapid process compared to development of new antibiotics which takes several years and require costly clinical trials prior to use (Weber-D et al., 2000). Furthermore, owing to the abundant and ubiquitous nature of bacteriophages, phages against the major pathogenic bacteria are readily discovered and isolated from environments that are habitats for host bacteria, especially from sewage, soil, water, and waste materials which contain high bacterial concentration, hence aiding in lowering the cost of production (Vinod et al., 2006; Skurnik et al., 2007). Phages are considered to have low environmental impact as they consist of nucleic acids and proteins only and have narrow host ranges (Loc-Carrillo and Abedon, 2011). In addition, phages can easily be applied as sprays or by directly mixing with water. In 2006, the Food and Drug Administration (FDA) approved a bacteriophage mixture, called a “lytic cocktail,” in a spray-on form designed to reduce the presence of *Listeria monocytogenes* bacteria in meat and deli products (Zach, 2010). For example, ListShield^TM^ (Intralytix, Inc.) is a commercial product marketed in a concentrated aqueous phage that is stored in 2–6°C. For direct food applications, the diluted working solution is typically applied directly on food surfaces by spraying at a concentration of approximately 1–2 mL per 250 square cm of food product surface. The recommended application rate for foods with complex surfaces is usually 1–4 mL of the diluted working solution per pounds of food. While, for environmental applications, the diluted working solution is typically applied onto the surfaces by spraying, or with a cloth, mop, or sponge, so that the targeted surface is thoroughly covered. About 50 mL of the diluted working solution is able to treat approximately 4 ft^2^ of surface.

Currently, Biologix, an Australian biotechnology company is developing phage therapy for *Vibrio* sp. associated with mortalities in the aquaculture. Jafral, an independent contract manufacturing organization (CMO) and contract research organization (CRO) located in Slovenia has been manufacturing bacteriophages. Here, bacteriophages has been successfully manufactured using manufacturing processes that have up to 10-time higher productivity. The end product can be used either in food industry or for animal and human treatments where it is desirable that phage titres are high and impurities levels are low.

The usage of antibiotics in the aquaculture industry has led to the increase of antibiotic resistant bacteria and development of ARGs in the environment which shade health risks to humans and animals (Kemper, 2008; Letchumanan et al., 2015a). Bacteriophages have the potential to reduce the dependency of aquaculture industry on use of antibiotics. The phages could be utilized instead of antibiotics to control bacterial infections that occur in aquaculture industry. Hence, plasmid mediated ARGs profile among bacteria would reduce when there is no antibiotics present in the environment. This eventually will preserve the ecosystem and reduce the effects on humans and animals.

In addition, bacteriophages have ability to disrupt bacterial biofilms (Azeredo and Sutherland, 2008). Bacteriophages have the capabilities to produce depolymerases which could hydrolyze extracellular polymers in bacterial biofilms. The use of phages were reported useful in the treatment of biofilm forming pathogens such as *Pseudomonas aeruginosa* (Fu et al., 2010), *Escherichia coli* (Doolittle et al., 1995), and *Staphylococcus aureus* (Sass and Bierbaum, 2007). It has been reported that around 80% of bacterial cases in the United States are associated with biofilms (Janssens et al., 2008). Biofilm has been a problematic disease in many food industries including seafood processing (Shikongo-Nambabi, 2011), dairy processing (Chmielewski and Frank, 2003), poultry processing (Harvey et al., 2007), and meat processing (Sofos and Geornaras, 2010). A study in USA has offered an insight into the potential use of phages to treat biofilm diseases by using an *in vitro* catheter model that was treated with phages. The results demonstrated that while phage treatment never fully prevented biofilm formation, biofilm biomass and cell density was significantly reduced (Curtin and Donlan, 2006; Fu et al., 2010). Luo et al. (2015) isolated two phages P4A and P4F which belong to the *Siphoviridae* family from seawater of an abalone farm and applied the phages to reduce *Vibrio* biofilm. In the study, the both phages were able to bring about 2 logs reduction in a *Vibrio harveyi* biofilm cell density after 24 h of phage treatment (Luo et al., 2015).

## Drawbacks of Phage Therapy

Despite all the listed advantages above, bacteriophage therapy does have its drawbacks. One of those that caused much concern is the potential emergence of phage-resistant bacteria, similar to that seen with antibiotic treatments. Typically, resistance would develop toward a particular phage when the bacterial surface proteins facilitating phage attachment are lost or lack of adsorption, thus preventing the phage from infecting its host. However, from the literature, the rate of developing resistance to phages is approximately 10-fold lower than to antibiotics (Tanji et al., 2004). Besides, other concern of *Vibrio* phage therapy is that some bacteriophage may be involved in the transfer of virulence genes to the bacteria. It was reported that toxicity of *Vibrio harveyi* to *Penaeus monodon* is induced by bacteriophage (Munro et al., 2003). Therefore, before using a bacteriophage for therapy it is important to test if they carry any virulence genes and would it be safe to use the bacteriophage (Vinod et al., 2006).

Another potential drawback on phage therapy is the bacterial defense system called CRISPR/Cas (clustered regularly interspaced short palindromic repeats). This CRISPR base immunity acts by integrating short virus sequences in the bacteria’s CRISPR locus, allowing the bacteria to recognize and clear infections. However, it has been demonstrated that this system can be utilized by the bacteriophages to promote infection. *Vibrio cholerae* ICP1 phages carry a Type I-F CRISPR–Cas system that targets a host locus, PLE, containing an anti-phage system. *Vibrio cholerae* ICP1 phage uses the CRISPR/Cas system to target the PLE for host cell destruction and successfully replicate. Due to bacteria cell death and DNA damage by lytic phage infection, CRISPR-mediated DNA cleavage of the PLE does not affect *Vibrio cholerae* ICP1 infection (Seed et al., 2013).

In addition, there is also a need to overcome the understandable stigma among consumers regarding safety of intentional consumption of viruses in spite of certification by the regulatory bodies. Additional work should be carried out in order to assess consumer knowledge and acceptance of phage therapy followed by targeted educational campaigns to raise awareness and acceptance.

## Conclusion

**FIGURE 1:**
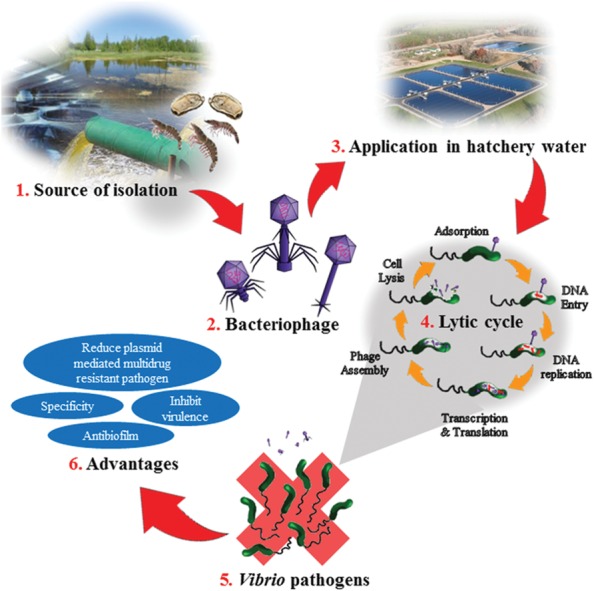
**Illustration on bacteriophage application in the aquaculture and the advantages.** (1) Bacteriophages are isolated from environmental sources such as hatcheries, sewage, ponds, or aquatic animals. (2) The isolated bacteriophage are purified and identified. The three common phages used as biocontrol agent for *Vibrio* sp; *Siphoviridae*, *Myoviridae*, and *Podoviridae*. (3) In the hatchery, the bacteriophage is added to the hatchery water. The amount of phage need to be added depends on size of the pond and amount of shrimps or cockles or fish in the pond. (4) Once added, the bacteria on host cell will undergo lytic cycle. Adsorption step would take place when the phages come in contact with the infected host cell. Then the phage’s DNA would penetrate into the host cell and replicate. It is followed by transcription and translation of the phage and DNA. Then the phage would assemble, host will lysis and phages will be released out from the host cell. (5) Bacteria such as *Vibrio* sp. would be eliminated from the hatchery. (6) Advantages of bacteriophages applications in a bacterial infection.

*Vibrio* species infection poses a threat in many fields, the treatment and control of which is currently dependent on antibiotic therapy; however, the use of antibiotics needs to be restricted due to the increase of antibiotic resistant bacteria (World Health Organization [WHO], 2006). Bacteriophage therapy is regarded as a highly viable alternative to prevent and control bacterial infections and in some conditions it has been proven to be superior to antibiotics. A schematic figure is been represented to illustrate the application of bacteriophage in the aquaculture and the advantages (**Figure 1**). The phages poses great advantages such as having host specificity, environmental friendly, readily discovered and isolated from the environment, and cost effective compared to antibiotics. Bacteriophages have the ability to control luminous vibriosis among *Vibrio* species (Vinod et al., 2006; Karunasagar et al., 2007). The phages have great potential as a bio-control agent to control and inhibit virulence of *Vibrio* species isolated from both clinical and environmental samples (Jassim and Limoges, 2014). In addition, it can be utilized in the agriculture and aquaculture industries instead of antibiotics to control bacterial infections that occur in aquaculture industry. This eventually will reduce the dependency toward antibiotics that leads to resistant genes profile in the environment (Golkar et al., 2014). Bacteriophages – being natural products are also generating less adverse effects compared to antibiotics. In 2006, US Food and Drug Administraion (FDA) approved the use of commercial phage cocktail ListShield^TM^ targeting *Listeria monocytogenes*. This is a confirmation that FDA has viewed phages are safe for human application and opens the doors for phage commercialization for human application and consumption (Housby and Mann, 2009). In March 2016, Intralytix, a biotechnology company received USDA, NIFA Phase II SBIR Grant to develop a phage based application to protect hatchery raised oysters from *Vibrio tubiashii* and *Vibrio coralliilyticus*. Moreover, there are many vibriophages that has been patented including phage patent number CN 103992990 A, CN 102524131 B, and US 20140105866 A1. The phage (US 20140105866 A1) is specific against *Vibrio anguillarum* was identified belonging to *Siphoviridae* family with a genome size of 48 kb. It possess prophylaxis properties, control and/or treatment of infection caused by *Vibrio anguillarum* in all types of species of fish, mollusks and crustaceans (Espejo et al., 2014). A lytic phage VP4B was reported to cause a significant growth inhibition effect of pathogenic *Vibrio harveyi*, and this patented phage can be used for biological prevention or control of vibrio diseases in mariculture (CN 103555671 A; Zhuhua et al., 2014). Qiu et al. (2012) reported a technique that utilize aquatic invertebrate larvae and adults to harmlessly carry *Vibrio* phages. The phages obtained through this technique are not virulent and can retain the lysis activity for host bacteria during a long period of time (CN 102550458 A). Jinyong et al. (2012) patent a phage BPH-VP-1 (CN 101798568 B) that exhibited broad lysis properties against *Vibrio parahaemolyticus*. It was reported that phage BPH-VP-I could be used alone or in combination, and as fungicides sprayed on food production plants in order to control *Vibrio parahaemolyticus* contamination. In summary, all the above listed advantages make bacteriophage therapy an attractive and promising tool as a biological control of bacterial infections.

## Author Contributions

VL and L-HL contributed to the literature database search, data collection, data extraction and writing of the manuscript. N-SA, PP, SS, AD, B-HG, K-GC and L-HL contributed vital insight and proofread on the writing. The research topic was conceptualized by L-HL.

## Conflict of Interest Statement

The authors declare that the research was conducted in the absence of any commercial or financial relationships that could be construed as a potential conflict of interest.
